# p16 Immunohistochemical Expression in Head and Neck Squamous Cell Carcinoma: Association With Prognostic Parameters

**DOI:** 10.7759/cureus.8601

**Published:** 2020-06-13

**Authors:** Atif A Hashmi, Naila Younus, Samreen Naz, Muhammad Irfan, Zubaida Hussain, Sara T Shaikh, Javaria Ali, Naveen Faridi, Javeria Najam, Maira Shoaib, Shumaila K Hashmi

**Affiliations:** 1 Pathology, Liaquat National Hospital and Medical College, Karachi, PAK; 2 Pathology, HITEC Institute of Medical Sciences, Taxila, PAK; 3 Epidemiology and Public Health, Liaquat National Hospital and Medical College, Karachi, PAK; 4 Medicine, Liaquat National Hospital and Medical College, Karachi, PAK; 5 Pathology, Combined Military Hospital (CMH) Multan Institute of Medical Sciences, Karachi, PAK

**Keywords:** head and neck squamous cell carcinoma, hpv, p16, oropharyngeal squamous cell carcinoma

## Abstract

Background

p16 is a tumor suppressor gene, over expression of which is considered as a surrogate marker of oncogenic human papillomavirus (HPV) infection. Moreover, p16 over expression correlates with good prognosis in head and neck squamous cell carcinoma (HNSCC). In the present study, we aimed to evaluate the frequency of p16 overexpression in HNSCC in our setup and its association with clinicopathologic parameters.

Methods

We performed p16 immunohistochemistry (IHC) on 144 cases of HNSCC. Association of p16 overexpression with various clinicopathologic parameters including T-stage, N-stage, grade, recurrence status, and risk factors was evaluated.

Results

p16 over expression was noted in 22.9% (33 cases), while 21.5% (31 cases) were focal positive and 55.6% (80 cases) were negative for p16 over expression. On the basis of percentage of expression; > 70% p16 expression was noted in 4.9% (7 cases), 9% (13 cases) showed 51% - 70% p16 expression, 9% (13 cases) revealed 11%-50% p16 expression, while 77.1% cases revealed no expression or < 10% p16 expression. Significant association of p16 expression was noted with nodal metastasis and extranodal spread while no significant association of p16 was noted with other prognostic parameters and risk factors.

Conclusion

Our data revealed that high expression (> 50%) of p16 is low in oropharyngeal squamous cell carcinoma in our setup. These finding suggest a low prevalence of HPV as a cause of HNSCC in our population. Moreover, p16 expression was found to be associated with some good prognostic parameters like lack of nodal metastasis, however, no significant association was noted with overall disease-free survival.

## Introduction

Head and neck squamous cell carcinoma (HNSCC) is the sixth most common cancer worldwide and is the significant cause of morbidity and mortality [1-3]. Traditional risk factors of HNSCC are alcohol and tobacco. On the other hand, in South-Asian countries like India and Pakistan, areca nut chewing (pan/gutka) is considered a more significant risk factor in the causation of this disease [4, 5]. However, a significant number of cases did not reveal exposure to these products. In western countries, incidence of oral squamous cell carcinoma (OSCC) is rising [6,7]. Infection with high-risk human papillomavirus (HPV) is asserted as the reason behind this arising incidence [8].

p16 is a tumor suppression gene, inactivation of which is considered as the major oncogenic event in the carcinogenesis of OSCC. Over expression of p16 is strongly associated with HPV infection and therefore immunohistochemical expression of p16 is considered as a surrogate marker of oncogenic HPV infection [9-11]. Moreover, p16 over expression is also considered a favorable prognostic marker in OSCC, as patients with p16 over expressing tumors have shown better diseases-free survival compared to tumors, which lack p16 expression [12,13]. However, neither frequency of p16 over expression in HNSCC in our setup has been widely studied, nor HPV infection in OSCC. Therefore, in the present study, we aim to evaluate the frequency of over expression in HNSCC of our setup and its association with various clinicopathological parameters.

## Materials and methods

The study was carried out at the Liaquat National Hospital, Karachi from January 2008 till December 2013. During this period of seven years, 144 patients who had surgeries for HNSCC were included in the study. An approval for conducting this study was taken from institutional ethical review and research committee. Informed written consent was taken from the patients prior to surgery. Hematoxylin and eosin stained slides of these cases were retrieved and independently re-evaluated by two surgical pathologists and pathological findings like tumor type and grade, tumor and nodal stage, perineural and lymphovascular invasion. History of addiction was recorded from hospital archives and last follow up was recorded. These clinical records were only available in 57 cases. Moreover, p16 immunohistochemistry (IHC) was performed on the representative tissue blocks of all cases.

**Figure 1:**
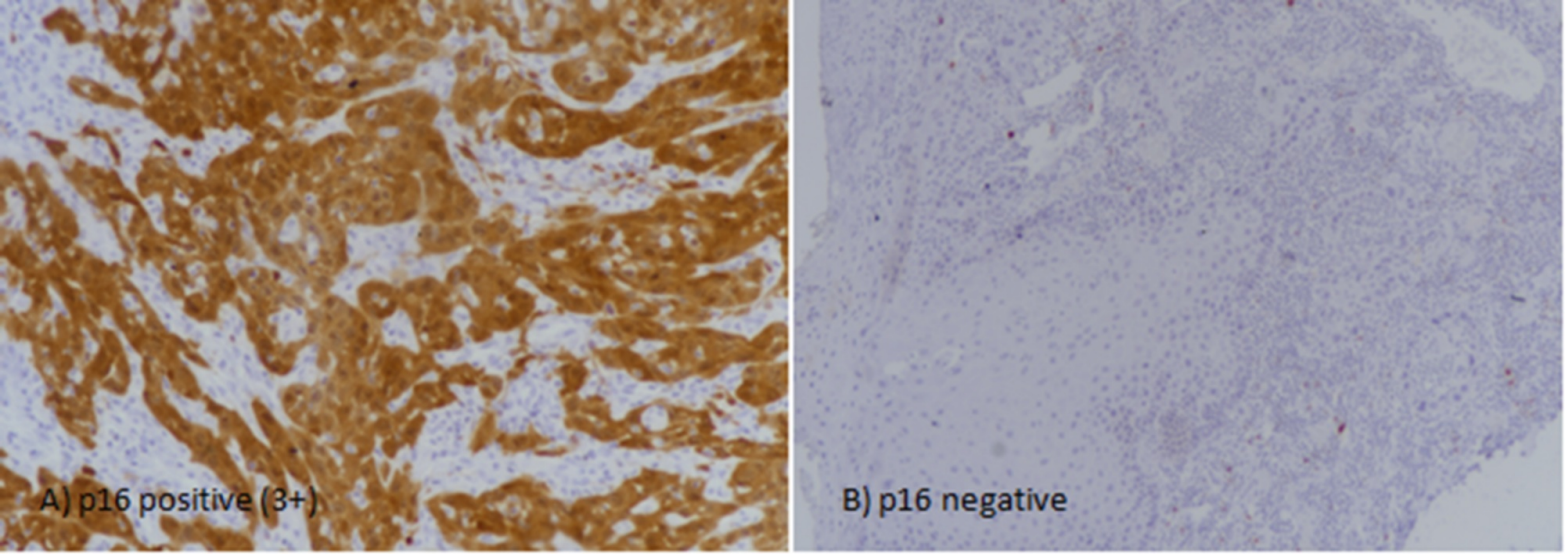
p16 expression in squamous cell carcinoma of the oral cavity

p16 antibody was purchased from Roche Ventana (Tucson, Arizona) and IHC was performed using antibody CINtec R p16INK4a, clone E6H4TM according to the manufacturers protocol. Tonsils and carcinoma cervix was taken as positive controls. Both nuclear and cytoplasmic staining was considered. Intensity of staining was divided into no staining (0), weak (1+), intermediate (2+), and strong (3+). On the other hand, the percentage of positively stained cancer cells was calculated as continuous variable. Intermediate to strong staining in >10% cancer cells was taken as positive while weak to intermediate staining in <10% cancer cells was considered focal positive (Figure 1). Similarly, p16 immunostaining was also assorted, according to the percentage of positive cells, into different groups.

Hospital medical records were reviewed and recurrence status was recorded. Overall survival was defined as time from surgical excision till death or last follow-up and disease-free survival was taken as the time between surgical excision and local recurrence or distant metastasis, last follow-up or death. Statistical package for social sciences, version 21 (SPSS, Chicago, IL) was used for data entry and analysis. Mean and standard deviation were calculated for categorical variables. Frequency and percentage were evaluated for qualitative variables. Chi-square was applied to determine association. Survival curves were plotted using Kaplan- Meier method and the significance of difference between survival curves were determined using log-rank ratio. P-value of ≤0.05 was taken as significant.

## Results

Patients mean age was 51.28+12.14 with 45.8% patients above 50 years. History of addiction especially pan/gutka was noted in 59.6% cases. Most common site of tumor was oral cavity (66.7%). 24.3% cases were at T1 stage, while 52.8% cases were found to be at N0 stage; 59% cases were keratinizing and 27% cases were of grade I. Lymphovascular and perineural invasion was noted in 1.4% and 13.9% cases, respectively and 56.9% of cases recurred after primary surgery as shown in Table 1.

**Table 1:** Association of p16 expression with clinicopathologic parameters of head and neck squamous cell carcinoma

	n(%)				P-Value
	Positive (n=33)	Negative (n=80)	Focal Positive (n=31)	Total (n=144)	
Age group					
≤30 years	0(0)	6(7.5)	0(0)	6(4.2)	0.325
31-50 years	16(48.5)	41(51.3)	15(48.4)	72(50)	
>50 years	17(51.5)	33(41.3)	16(51.6)	66(45.8)	
Gender					
Male	23(69.7)	59(73.8)	24(77.4)	106(73.6)	0.782
Female	10(30.3)	21(26.3)	7(22.6)	38(26.4)	
History of pan(n=57)					
Yes	6(66.7)	19(54.3)	9(69.2)	34(59.6)	0.685
No	3(33.3)	16(45.7)	4(30.8)	23(40.4)	
History of smoking(n=57)					
Yes	0(0)	3(8.6)	1(7.7)	4(7)	1.000
No	9(100)	32(91.4)	12(92.3)	53(93)	
History of alcohol(n=57)					
Yes	0(0)	1(2.9)	0(0)	1(1.8)	1.000
No	9(100)	34(97.1)	13(100)	56(98.2)	
Location of tumor					
oral cavity	24(72.7)	53(66.3)	10(61.3)	96(66.7)	0.272
lip	1(3)	0(0)	2(6.5)	3(2.1)	
tongue	6(18.2)	24(30)	9(29)	39(27.1)	
soft palate	2(6.1)	3(3.8)	1(3.2)	6(4.2)	
Tumor stage					
T1	9(27.3)	16(20)	10(32.3)	35(24.3)	0.168
T	20(60.6)	38(47.5)	14(45.2)	72(50)	
T3/T4	4(12.1)	26(32.5)	7(22.6)	37(25.7)	
Depth of invasion					
<2cm	29(87.9)	64(80)	28(90.3)	121(84)	0.394
≥2cm	4(12.1)	16(20)	3(9.7)	23(16)	
Nodal stage					
No	25(75.8)	36(45)	15(48.4)	76(52.8)	0.001
N1	0(0)	11(13.8)	9(29)	20(13.9)	
N2a	0(0)	0(0)	0(0)	0(0)	
N2b	7(21.2)	31(38.8)	5(16.1)	43(29.9)	
N2c	1(3)	1(1.3)	2(6.5)	4(2.8)	
N3	0(0)	1(1.3)	0(0)	1(0.7)	
Extranodal extention					
Not Present	30(90.9)	54(67.5)	25(80.6)	109(75.7)	0.024
Present	3(9.1)	26(32.5)	6(19.4)	35(24.3)	
Histological subtypes					
Non-keratinizing	4(12.1)	14(17.5)	2(6.5)	20(13.9)	0.639
Keratinizing	21(63.6)	45(56.3)	19(61.3)	85(59)	
Non-keratinizing with maturation	8(24.2)	21(26.3)	10(32.3)	39(27.1)	
Histologic grade					
Grade-I	11(33.3)	16(20)	12(38.7)	39(27.1)	0.234
Grade-II	20(60.6)	53(66.3)	16(51.6)	89(61.8)	
Grade-III	2(6.1)	11(13.8)	3(9.7)	16(11.1)	
Lymphovascular invasion					
Not Present	33(100)	78(97.5)	31(100)	142(98.6)	1.000
Present	0(0)	2(2.5)	0(0)	2(1.4)	
Perineural invasion					
Not Present	30(90.9)	68(85)	26(83.9)	124(86.1)	0.687
Present	3(9.1)	12(15)	5(16.1)	20(13.9)	
Recurrence (n=58)					
Yes	6(66.7)	20(55.6)	7(53.8)	33(56.9)	0.808
No	3(33.3)	16(44.4)	6(46.2)	25(43.1)	
Chi Square test applied.					
P-value≤0.05 considered as significant.					

p16 over expression was noted in 22.9% (33 cases), while 21.5% (31 cases) were focal positive and 55.6% (80 cases) were negative for p16 over expression. On the basis of percentage of expression; > 70% p16 expression was noted in 4.9% (7 cases), 9% (13 cases) showed 51% - 70% p16 expression, 9% (13 cases) revealed 11%- 50% p16 expression, while 77.1% cases revealed no expression or < 10% p16 expression. Significant association of p16 expression was noted with nodal metastasis and extranodal spread while no significant association of p16 was noted with other prognostic parameters and risk factors as shown in Table 1 and Table 2.

**Table 2:** Association of p16 expression categories with clinicopathologic parameters of head and neck squamous cell carcinoma

	n(%)					P-Value
	≤10% (n=111)	11-50% (n=13)	51-70% (n=13)	>70% (n=7)	Total (n=144)	
Age group						
≤30 years	6(5.4)	0(0)	0(0)	0(0)	6(4.2)	0.793
31-50 years	56(50.5)	8(61.5)	6(46.2)	2(28.6)	72(50)	
>50 years	49(44.1)	5(38.5)	7(53.8)	5(71.4)	66(45.8)	
Gender						
Male	83(74.8)	7(53.8)	10(76.9)	6(85.7)	106(73.6)	0.422
Female	28(25.2)	6(46.2)	3(23.1)	1(14.3)	38(26.4)	
History of pan(n=57)						
Yes	28(58.3)	1(50)	4(66.7)	1(100)	34(59.6)	1.000
No	20(41.7)	1(50)	2(33.3)	0(0)	23(40.4)	
History of smoking(n=57)						
Yes	4(8.3)	0(0)	0(0)	0(0)	4(7)	1.000
No	44(91.7)	2(100)	6(100)	1(100)	53(93)	
History of alcohol(n=57)						
Yes	1(2.1)	0(0)	0(0)	0(0)	1(1.8)	1.000
No	47(97.9)	2(100)	6(100)	1(100)	56(98.2)	
Location of tumor						
oral cavity	72(64.9)	10(76.9)	9(69.2)	5(71.4)	96(66.7)	0.243
lip	2(1.8)	0(0)	0(0)	1(14.3)	3(2.1)	
tongue	33(29.7)	2(15.4)	4(30.8)	0(0)	39(27.1)	
soft palate	4(3.6)	1(7.7)	0(0)	1(14.3)	6(4.2)	
Tumor stage						
T1	26(23.4)	6(46.2)	3(23.1)	0(0)	35(24.3)	0.076
T2	52(46.8)	7(53.8)	7(53.8)	6(85.7)	72(50)	
T3/T4	33(29.7)	0(0)	3(23.1)	1(14.3)	37(25.7)	
Tumor depth						
<2cm	92(82.9)	11(84.6)	11(84.6)	7(100)	121(84)	0.846
≥2cm	19(17.1)	2(15.4)	2(15.4)	0(0)	23(16)	
Nodal Stage						
No	51(45.9)	8(61.5)	11(84.6)	6(85.7)	76(52.8)	0.069
N1	20(18)	0(0)	0(0)	0(0)	0(0)	
N2a	0(0)	0(0)	0(0)	0(0)	0(0)	
N2b	36(32.4)	5(38.5)	2(15.4)	0(0)	43(29.9)	
N2c	3(2.7)	0(0)	0(0)	1(14.3)	4(2.8)	
N3	1(0.9)	0(0)	0(0)	0(0)	0(0)	
Extranodal extention						
Not Present	79(71.2)	12(92.3)	12(92.3)	6(85.7)	109(75.7)	0.164
Present	32(28.8)	1(7.7)	1(7.7)	1(14.3)	35(24.3)	
Histological subtypes						
Non-keratinizing	16(14.4)	0(0)	2(15.4)	2(28.6)	20(13.9)	0.327
Keratinizing	64(57.7)	10(76.9)	9(69.2)	2(28.6)	85(59)	
Non-keratinizing with maturation	31(27.9)	3(23.1)	2(15.4)	3(42.9)	39(27.1)	
Histologic grade						
Grade-I	28(25.2)	2(15.4)	8(61.5)	1(14.3)	39(27.1)	0.073
Grade-II	69(62.2)	11(84.6)	4(30.8)	5(71.4)	89(61.8)	
Grade-III	14(12.6)	0(0)	1(7.7)	1(14.3)	16(11.1)	
Lymphovascular invasion						
Not Present	109(98.2)	13(100)	13(100)	7(100)	142(98.6)	1.000
Present	2(1.8)	0(0)	0(0)	0(0)	2(1.4)	
Perineural invasion						
Not Present	94(84.7)	12(92.3)	12(92.3)	6(85.7)	124(86.1)	0.885
Present	17(15.3)	1(7.7)	1(7.7)	1(14.3)	20(13.9)	
Chi square test applied.						
P-Value≤0.05 considered as significant.						

**Figure 2:**
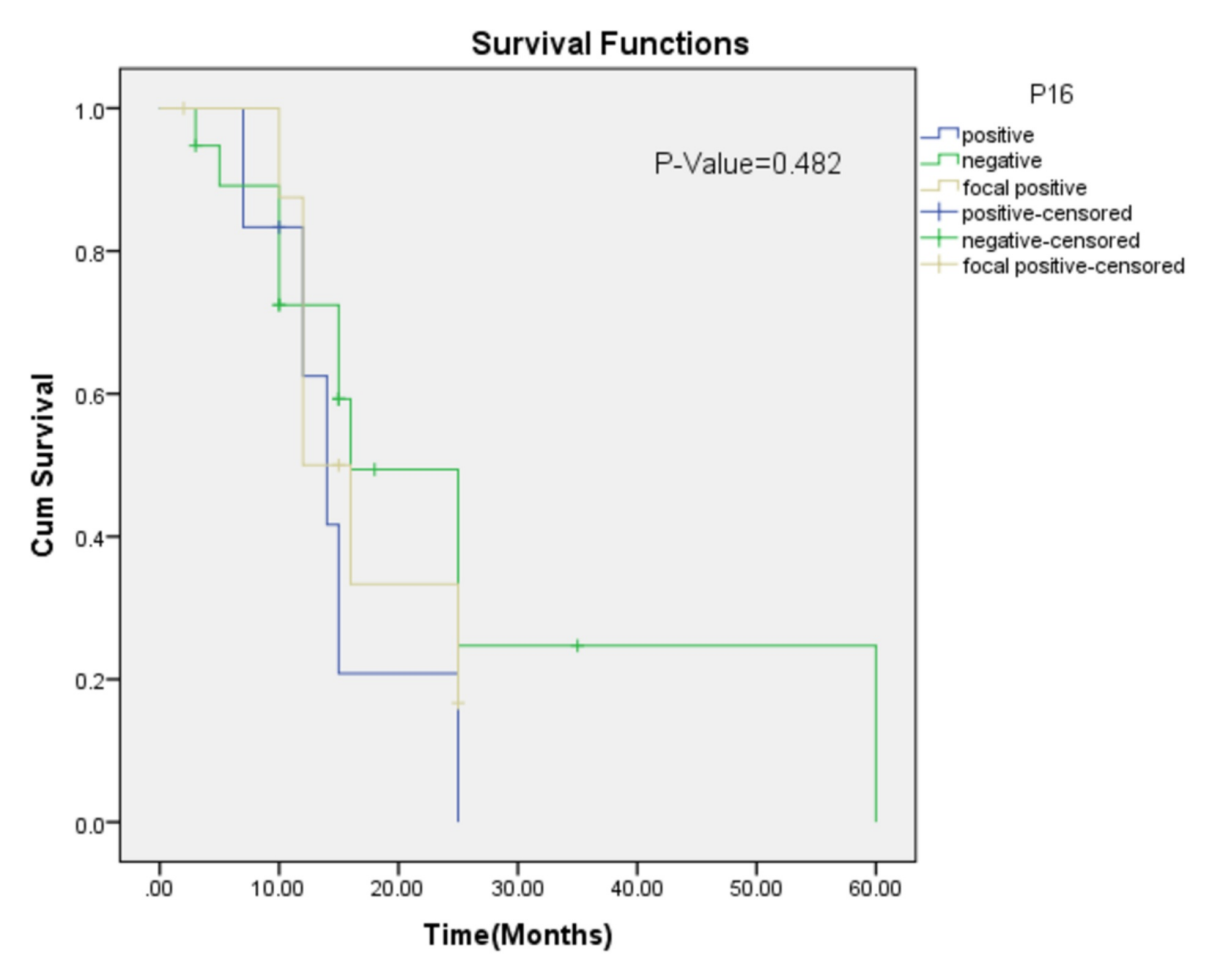
Kalpien-Meier for epidermal growth factor receptor (EGFR) overexpression (disease-free survival)

Similarly, no significant association was noted with recurrence status of the patients as presented in Figure 2.

## Discussion

In the present study, we evaluated p16 expression in HNSCC, as it is considered as the marker of HPV infection. Overall, p16 expression was 22.9% while high p16 expression i.e. 51% -70% and >70% expression was noted in only 9% and 4.9% cases, respectively.

Literature review revealed that p16 over expression strongly correlates with HPV infection. In a recent study, the authors compared the results of HPV-16 in situ hybridization and p16 IHC in 256 cases of HNSCC; 71.2% cases were HPV-16 positive and all cases showed p16 staining. They reported sensitivity, specificity, positive predictive value, and negative predictive value of p16 in relation to HPV infection as 100%, 74%, 9%, and 100%, respectively [14]. It is now widely accepted that HPV infection is the underlying cause of the rising incidence of OSCC. The HPV infection in patients with OSCC considerably varies in different areas of the world. A systemic review/meta-analysis of 148 studies, including 12,163 cases of HNSCC, revealed the prevalence rate of HPV in the oropharyngeal and oral cavity SCC as 45.8% and 24.2%, respectively [15]. Another systemic review assessed 3,680 cases of HNSCC - HPV DNA was detected in 24.9% of cases of oropharyngeal SCC [16]. Moreover, studies have reviewed that percentage of cancer cells staining with p16 along with confluent staining (back to back cell staining) and intensity of staining are also a parameter to be determined. Lewis et al. reported that only greater than 75% p16 staining or alternatively less than 50% staining combined with > 25% confluent areas is a suitable cut off for defining HPV infectivity [17]. Similarly, Larsen et al. proposed a cut off of >70% p16 staining (both nuclear and cytoplasmic) to predict the presence of transcriptionally active HPV [18]. We only found 50%-70% and> 70% p16 staining in 9% and 4.9% cases, respectively with strong staining intensity. These findings showed that HPV prevalence is low in our case of OSCC. p16 expression has an independent prognostic value in HNSCC. Association of p16 over expression with lower T-stage [19] and better survival has been reported [20]. We also found a significant association of p16 over expression with lower nodal stage, which is one of the most important prognostic factors in HNSCC.

Various studies evaluated impact of p16 expression in HNSCC along with its association with prognostic and pathological parameters. A meta-analysis found that p16 expression was associated with better clinical outcome and good prognostic factors including low risk of nodal metastasis [21]. Among other histologic parameters, p16 expression was noted to be associated with non-keratinizing phenotype [22], however, we did not find any such association in our study.

The major limitation of the study was that molecular studies were not performed to correlate IHC expression with the presence of HPV strains in cases of HNSCC; therefore, we recommend molecular testing of HPV in cases of HNCC especially in those with high p16 expression.

## Conclusions

Our data revealed that high expression (> 50%) of p16 is low in OSCC in our setup. These finding suggest a low prevalence of HPV as a cause of OSCC in our population. Moreover, p16 expression was found to be associated with some good prognostic parameters like lack of nodal metastasis; however, no significant association was noted with overall disease-free survival. 
